# Effect of advanced nursing care on psychological disorder in hypertensive retinopathy of pregnancy: A protocol of systematic review

**DOI:** 10.1097/MD.0000000000023970

**Published:** 2021-02-12

**Authors:** Xiao-fang Wang, Lin-juan Liu

**Affiliations:** a Department of Ophthalmology, Hanzhong 3201 Hospital, Hanzhong; b Second Ward of Obstetrics and Gynecology Department, Yan’an People's Hospital, Yan’an, Shaanxi, China.

**Keywords:** anxiety, depression, hypertensive retinopathy, nursing intervention, pregnancy

## Abstract

**Background:**

This study will assess the effect of advanced nursing care (ANC) on psychological disorder (PD) in hypertensive retinopathy of pregnancy (HTRP).

**Methods:**

This study will search electronic databases from inception to the present (Cochrane Library, MEDLINE, EMBASE, CINAHL, AMED, PsycINFO, CNKI, and Chinese Biomedical Literature Database), and other sources. All literature sources will be searched without limitations to language and study status. All eligible case-controlled study (CCS) will be included in this study. Two authors will independently carry out literature selection, data collection, and study quality assessment. Any confusion will be solved by a third author through discussion. Statistical analysis will be conducted using RevMan 5.3 software. In addition, a narrative synthesis will be elaborated if it is necessary.

**Results:**

This study will summarize most recent high quality evidence to appraise the effect of ANC on PD in HTRP.

**Conclusion:**

The results of this study will seek to identify the effect of ANC on PD in HTRP among pregnancy population.

**OSF registration:**

osf.io/hgp93.

## 1 Introduction

Hypertensive retinopathy is a common eye disorder,^[1–3]^ which occurs when the retinal vessels damaged because of the high blood pressure.^[4–7]^ It is usually asymptomatic and is diagnosed on fundoscopic features, but often manifests as signs of arteriovenous crossing, arterial, retinal, macular, and optic nerve changes.^[8,9]^ Its incidence ranges from 66.3% to 83.6% according to the different study reports.^[6,10,11]^ Several risk factors are associated with this condition, such as genetic factor, smoking, and plasma leptin.^[12–15]^ It not only affects general population with hypertension, but also occurs in pregnancy (also known as hypertensive retinopathy of pregnancy, HTRP), which results in high risk for both mother and fetus.^[16–18]^ In addition, many patients with HTRP often experience psychological disorder (PD), such as depression and anxiety.^[19,20]^ Thus, it is very important to manage this disorder as early as possible.

Advanced nursing care (ANC) is reported to effectively manage PD in patients with HTRP.^[19–25]^ Although several clinical studies reported its effect on PD in HTRP, no systematic review specifically addresses this topic. Thus, in this study, we will comprehensively search electronic databases and gray literature sources for trials that have investigated the benefits of ANC on PD in patients with HTRP.

## 2 Methods

### 2.1 Study registration

This study has been registered on OSF (osf.io/hgp93). We have reported this study according to the guidelines of Preferred Reporting Items for Systematic Reviews and Meta-Analysis Protocol Statement.^[26]^

### 2.2 Ethics and dissemination

This study will not need ethics approval, because it will not collect individual patient data. We will disseminate this study on a peer-reviewed journal or a conference meeting.

### 2.3 Study eligibility criteria

#### 2.3.1 Types of studies

This study will consider case-controlled study (CCS) on investigating the effect of ANC on PD in HTRP. We will exclude all non-clinical study and uncontrolled study.

#### 2.3.2 Types of participants

All eligible female adults (≥18 years old) who were diagnosed as PD in HTRP will be included in this study. No limitations will be applied to their race and educational background.

#### 2.3.3 Types of interventions

##### 2.3.3.1 Intervention

All patients in the experimental group received any types of ANC on PD in HTRP.

##### 2.3.3.2 Comparator

Eligible comparators include any management, such as medication, routine nursing care, or no treatment.

#### 2.3.4 Type of outcome measurements

##### 2.3.4.1 Primary outcomes

Depression (measured by any associated scale, such as Self-Rating Depression Scale); Anxiety (measured by any relevant tool, such as Self-Rating Anxiety Scale).

##### 2.3.4.2 Secondary outcomes

Insomnia (assessed by any related scale, such as Pittzburg Insomnia Quality Scale);

Quality of life (appraised by any tool, such as 36-Item Short Form Health Survey); Adverse events.

### 2.4 Search strategy and data management

#### 2.4.1 Search strategy

We will handle literature search from electronic databases from inception to the present (Cochrane Library, MEDLINE, EMBASE, CINAHL, AMED, PsycINFO, CNKI, and Chinese Biomedical Literature Database) and other sources (such as conference abstracts, and reference lists of included studies) involving the use of ANC on PD in HTRP. The search strategy will be built in consultation by an experienced medical information specialist. No limitations of language and study status will be imposed to this study. A detailed search strategy of Cochrane Library is shown in Table 1. We will also adapt and modify similar search strategy to other electronic databases.

**Table 1:** Search strategy of Cochrane Library.

Number	Search terms
1	MeSH descriptor: (hypertensive retinopathy) explode all trees
2	MeSH descriptor: (pregnancy) explode all trees
3	((hypertension∗) or (hypertensive∗) or (blood pressure∗) or (retinopathy∗) or (retinal vessel∗) or (optic neuropathy∗) or (pregnancy∗) or (pregnant∗)):ti, ab, kw
4	Or 1–3
5	MeSH descriptor: (depression) explode all trees
6	MeSH descriptor: (anxiety) explode all trees
7	((psychological∗) or (depressive∗) or (anxiety∗) or (pressure∗) or (disorder∗) or (nervous∗) or (mood∗)):ti, ab, kw
8	Or 5–7
9	(nursing care) explode all trees
10	((nursing∗) or (care∗) or (long term∗) or (short term∗) or (health care∗) or (high quality∗) or (advanced∗) or (consultation∗)):ti, ab, kw
11	Or 9-10
12	MeSH descriptor: (randomized controlled trials) explode all trees
13	((random∗) or (randomly∗) or (placebo∗) or (blind∗) or (allocation∗) or (control∗) or (clinical∗)or (trial∗) or (study∗)):ti, ab, kw
14	Or 12–13
15	4 and 8 and 11 and 14

#### 2.4.2 Study selection

Two independent authors will scan the title/abstract of each searched citation, and we will remove all irrelevant studies. In addition, the full text of any article will be judged against all inclusion criteria. Any disagreement on study selection will be settled down by discussion with the help of a third author. The whole process of study selection will be presented in a flow chart.

#### 2.4.3 Data extraction and management

All necessary data will be collected by 2 independent authors using a standardized template form. We will invite a third author to solve any divergence between 2 authors. Data will be collected from eligible studies: study characteristics (e.g., first author, time of publication, country), patient information (e.g., race, age, condition severity), intervention and comparator (e.g., treatment types, dosage, frequency), outcomes (e.g., primary and secondary outcome measurements, follow-up information), conflict of interest, and funding information.

#### 2.4.4 Dealing with missing data

Any insufficient, unclear, or missing data will be obtained by contacting original authors through email or fax. We will analyze available data only if those data are not achieved.

### 2.5 Study quality assessment

Two authors will independently appraise study quality for each eligible study. We will assess study quality using Newcastle–Ottawa Scale.^[27]^ Any difference regarding the study quality assessment will be solved by a third author through discussion.

### 2.6 Statistical analysis

In this study, we will conduct statistical analysis using RevMan 5.3 software (Cochrane Community, London, UK). We will express dichotomous values as risk ratio and 95% confidence intervals (CIs), and continuous values as mean difference (MD) or standardized MD and 95% CIs. Statistical heterogeneity across included studies will be evaluated by *I*^2^ test. *I*^2^ ≤ 50% suggests low level of heterogeneity and we will employ a fixed-effect model for data pooling. *I*^2^ > 50% indicates high level of heterogeneity, and we will use a random-effect model for data synthesis. If data are sufficient and adequately similar on the same outcome with low level of heterogeneity, meta-analysis will be conducted. If there are insufficient data, meta-analysis will not be performed. We will carry out subgroup analysis if there is high level of heterogeneity. In addition, we will report descriptive summaries for study results.

### 2.7 Additional analysis

Subgroup analysis will be employed based on different study characteristics, patient demographics, study types, study methods, and interventions and controls. If sufficient data are available, sensitivity analysis will be carried out to explore the stability of merged outcome data by removing low quality study. Reporting bias will be examined by funnel plot and Egger regression test if over 10 studies are included.

## 3 Discussion

PD (including depression and anxiety) is a common issue in patients with HTRP. Interest in the use of ANC as a management for this disorder has developed over the past decade. Several studies have reported to utilize ANC for the treatment of PD in HTRP. However, there is no relevant systematic review specifically investigating this topic.

The present study firstly yields high quality evidence on the effect of ANC for the treatment of PD in HTRP. It will provide a comprehensive summary of the current evidence of ANC on PD in patients with HTRP and will merge new evidence as it is available. Its results will provide solid data and robust evidence of ANC on PD in HTRP either for the clinical practice or for health policy makers.

## Author contributions

**Conceptualization:** Xiao-fang Wang, Lin-juan Liu.

**Data curation:** Xiao-fang Wang, Lin-juan Liu.

**Formal analysis:** Xiao-fang Wang, Lin-juan Liu.

**Investigation:** Lin-juan Liu.

**Methodology:** Xiao-fang Wang, Lin-juan Liu.

**Project administration:** Lin-juan Liu.

**Resources:** Xiao-fang Wang, Lin-juan Liu.

**Software:** Xiao-fang Wang, Lin-juan Liu.

**Supervision:** Lin-juan Liu.

**Validation:** Xiao-fang Wang, Lin-juan Liu.

**Visualization:** Xiao-fang Wang, Lin-juan Liu.

**Writing – original draft:** Xiao-fang Wang, Lin-juan Liu.

**Writing – review & editing:** Xiao-fang Wang, Lin-juan Liu.

## Abbreviations

Abbreviations: ANC = advanced nursing care, CCS = case-controlled study, CIs = confidence intervals, HTRP = hypertensive retinopathy of pregnancy, MD = mean difference, PD = psychological disorder.
